# Non-Invasive monitoring of diaphragmatic timing by means of surface contact sensors: An experimental study in dogs

**DOI:** 10.1186/1471-2466-4-8

**Published:** 2004-09-08

**Authors:** José Antonio Fiz, Raimon Jané, Abel Torres, Josep Morera, Batxi Galdiz, Joaquín Gea, Alejandro Grassino

**Affiliations:** 1 Servicio de Neumología, Hospital Universitario Germans Trias i Pujol, Badalona, Barcelona, Spain; 2 Dept. ESAII, Centre de Recerca en Enginyeria Biomèdica, UPC, Barcelona, Spain; 3 Servicio de Neumología, Hospital Cruces, Baracaldo, Bilbao, Spain; 4 Servicio de Neumología, Hospital del Mar, Barcelona, Spain; 5 Dept. Medicine, Notre Dame Hospital, Univ. Montreal, Montreal, Quebec, Canada

## Abstract

**Background:**

Non-invasive monitoring of respiratory muscle function is an area of increasing research interest, resulting in the appearance of new monitoring devices, one of these being piezoelectric contact sensors. The present study was designed to test whether the use of piezoelectric contact (non-invasive) sensors could be useful in respiratory monitoring, in particular in measuring the timing of diaphragmatic contraction.

**Methods:**

Experiments were performed in an animal model: three pentobarbital anesthetized mongrel dogs. The motion of the thoracic cage was acquired by means of a piezoelectric contact sensor placed on the costal wall. This signal is compared with direct measurements of the diaphragmatic muscle length, made by sonomicrometry. Furthermore, to assess the diaphragmatic function other respiratory signals were acquired: respiratory airflow and transdiaphragmatic pressure. Diaphragm contraction time was estimated with these four signals. Using diaphragm length signal as reference, contraction times estimated with the other three signals were compared with the contraction time estimated with diaphragm length signal.

**Results:**

The contraction time estimated with the TM signal tends to give a reading 0.06 seconds lower than the measure made with the DL signal (-0.21 and 0.00 for FL and DP signals, respectively), with a standard deviation of 0.05 seconds (0.08 and 0.06 for FL and DP signals, respectively). Correlation coefficients indicated a close link between time contraction estimated with TM signal and contraction time estimated with DL signal (a Pearson correlation coefficient of 0.98, a reliability coefficient of 0.95, a slope of 1.01 and a Spearman's rank-order coefficient of 0.98). In general, correlation coefficients and mean and standard deviation of the difference were better in the inspiratory load respiratory test than in spontaneous ventilation tests.

**Conclusion:**

The technique presented in this work provides a non-invasive method to assess the timing of diaphragmatic contraction in canines, using a piezoelectric contact sensor placed on the costal wall.

## Background

Non-invasive monitoring of respiratory function is an area of increasing research interest, resulting in the appearance of new monitoring devices [1]. At present, the most utilised non-invasive method for continuous quantitative monitoring of breathing pattern is respiratory inductive plethysmography. This technique allows the study of various breathing pattern parameters such as respiratory frequency, but it is based on an averaged measurement of the whole thoracic-abdominal movement. On the other hand, the use of mouthpieces or face masks in pneumotacography, influences in the tidal volume and respiratory frequency [2-4]. Other systems like piezoelectric contact sensors measure the system acceleration when placed on body surfaces. In previous works we have shown that the beginning and end of diaphragmatic contraction can be determined by inflexion points in the thoracic cage motion signal acquired with a contact sensor [5-7].

The purpose of this study is to evaluate a non-invasive method to study the timing of the diaphragmatic function, using an animal model (dogs). Accordingly, the present study was designed to test whether the use of contact (non-invasive) piezoelectric sensors, placed on the dogs' costal wall, could be useful in monitoring the diaphragm contraction period in different respiratory conditions, comparing it with other physiological signals such as transdiaphragmatic pressure, diaphragm length measured by sonomicrometry, and respìratory airflow. Diaphragm contraction time is expected to be very close to inspiratory time, which is one of the most utilized parameters in the studies of breathing pattern under experimental or clinical conditions.

## Methods

Three mongrel dogs (15–20 kg) were surgically instrumented under general anesthesia given via a femoral vein catheter (pentobarbital sodium, 25 mg/kg). Respiratory flow was recorded with a Fleisch pneumotachograph. Diaphragm shortening was measured via two piezoelectric crystals (Sonomicrometer, Triton Tech. Inc., m. 120), as described in [8]. The diaphragm was exposed by a midline abdominal incision, and the two piezoelectric crystals were implanted along the rib diaphragm fibres. An anterior midline incision was made in the neck to allow the left C5 and C6 phrenic nerve roots to be isolated. Motion of the thoracic cage surface was recorded by a piezoelectric contact sensor (HP 21050A) positioned on the costal wall and fixed to the skin by an elastic band. Maximal deflection of the accelerometer following a unilateral phrenic nerve electrical pulse was measured on the 6–7 intercostal space area of the rib cage, where the diaphragmatic fibres are directly apposed to its inner surface, thereby minimizing the distance between the accelerometer and the muscle. Transdiaphragmatic pressure was measured in the usual way as the difference between gastric and esophageal pressures, each recorded with the conventional balloon-catheter technique [9]. The electromyogram of the diaphragm (EMGdi) was recorded with two (parallel) 10 mm long single filament copper wires (1 mm in diameter) attached 20 mm apart on a semi-rigid plastic plate, as described in [10]. Measurements were made at a similar level of anaesthesia (corneal reflex just suppressed). All animals were in supine position during the study, and spinal anaesthesia was applied as a means to isolate diaphragmatic function by eliminating the activity of the intercostal muscles. To that effect, with the animal lying down with its head and neck raised, a hyperbaric Tetracaine solution (Sigma) was injected into the subarachnoid space at the lumbar level (bolus of 1 ml). The injection of tetracaine was halted when intercostals EMG activity was abolished below intercostals spaces 3–4 on both sides of the cage (as determined by needle electrodes recording from the parasternals). With the head and neck elevated, the animal was then turned over to the supine position. All dogs performed spontaneous ventilation before the use of spinal anaesthesia (SVN), and spontaneous ventilation (SVW) and respiration with an inspiratory resistive load (ILW) with spinal anaesthesia. The duration and respiratory frequency of the tests varies in function of the dog analyzed. Table I shows the number of cycles and the duration of the respiratory tests performed by the three dogs.

**Table 1:** Number of cycles and duration of the respiratory tests in the three dogs.

	**Spontaneous ventilations without anaesthesia (SVN)**		**Spontaneous ventilations with anaesthesia (SVW)**		**Inspiratory load with anaesthesia (ILW)**	
	
	**No. cycles**	**Duration (s)**	**No. cycles**	**Duration (s)**	**No. cycles**	**Duration (s)**
**DOG1**	45	180	40	160	159	400
**DOG2**	19	180	29	140	194	600
**DOG3**	11	60	8	30	46	220

**Figure 1:**
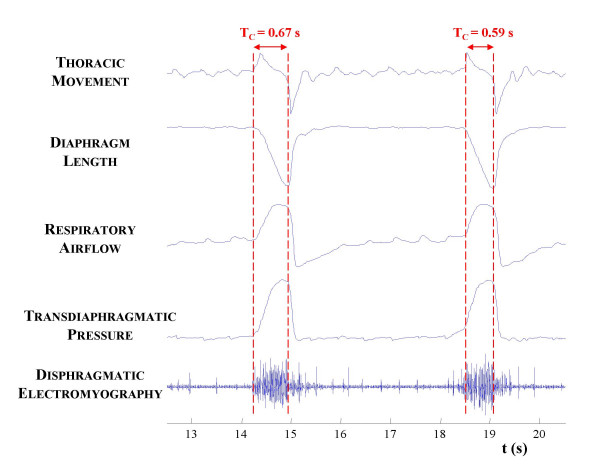
**Respiratory Signals. **Example of the five signals acquired during 8 seconds (two respiratory cycles) during the inspiratory load with spinal anaesthesia (ILW) respiratory test of the second dog.

All analogical signals were amplified (HP 8802A), filtered and digitised with a 12 bit A/D system at a sampling rate of 4 kHz. Inspiratory airflow (FL), diaphragm length (DL), thoracic cage motion (TM), transdiaphragmatic pressure (DP) and diaphragmatic electromyography (EMGdi) signals were decimated at a new sampling rate (FL, DL, DP: 100 Hz ; TM: 200 Hz; EMGdi: 1200 Hz) and digitally filtered (FL, DL, DP: DC-40 Hz ; TM: DC-80 Hz; EMGdi: 10–480 Hz). The sampling frequencies and filter bands were selected to be adapted to the frequency content of the signals. Figure 1 shows a typical strip-chart recording of the five signal acquired, during 8 seconds (2 respiratory cycles) of the ILW respiratory test of the second dog.

**Figure 2:**
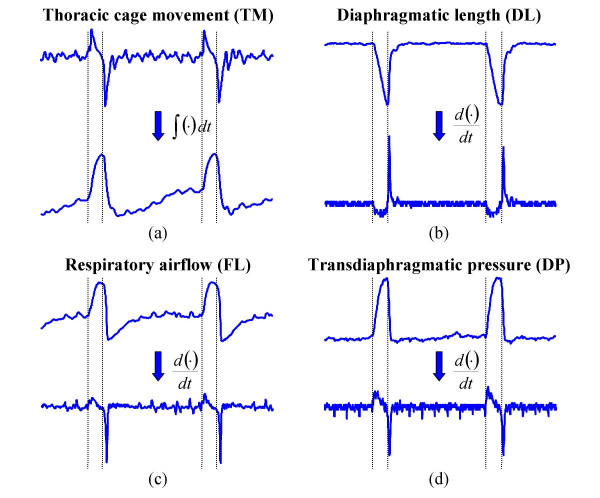
**Diaphragmatic contraction time detection. **Morphologies of thoracic cage motion signal and its integral (a), diaphragmatic length (b), respiratory airflow (c) and transdiaphragmatic pressure (d) and their first derivatives. Vertical lines mark initial and final instants of diaphragmatic contraction.

In order to detect the initial and final diaphragm contraction times (*t*_*i*_, *t*_*f*_), the integral of TM signal and the first derivative of the DL, FL and DP signals were computed. Initial contraction time is detected when these signals reach 10 % of the maximum. In a similar way, final contraction time is detected when the signals reach 10 % of their minimum. We also computed contraction period (*T*_*C *_= *t*_*f *_- *t*_*i*_). The EMGdi signal was included initially in the study but later was rejected because the postinspiratory activity present in this signal hindered the detection of the end of the diaphragm shortening contraction time (as seen in Fig. 1). One representative experimental record of TM, DL, FL and DP signals is shown in Fig. 2. Furthermore, the integral of the TM signal is presented, as well as the first derivative of the DL, FL and DP signals.

### Statistical Analysis

Differences between contraction periods obtained with DL signal and the other three signals were summarized by the mean (MEAN), standard deviation (STD). DL signal was used as a goldstandard (reference signal) for muscle shortening, since it is a direct measure of the reduction of the diaphragm muscle length, contrary to flow and trandiaphragmatic pressure which are more remote markers of muscle contraction.

Relationships between contraction periods obtained by means DL and TM signals, DL and FL, and DL and DP were analyzed by diverse statistical methods. Different correlation coefficient were calculated: the Pearson correlation coefficient (r), the intra-class correlation coefficient (or reliability coefficient: R) [11], the slope of the linear regression line (p), and the Spearman's rank-order correlation coefficient (r). Furthermore the Bland-Altman method for agreement analysis was performed [12]; in this graphical method the differences between two measures or techniques are plotted against the averages of the two techniques.

## Results

**Figure 3:**
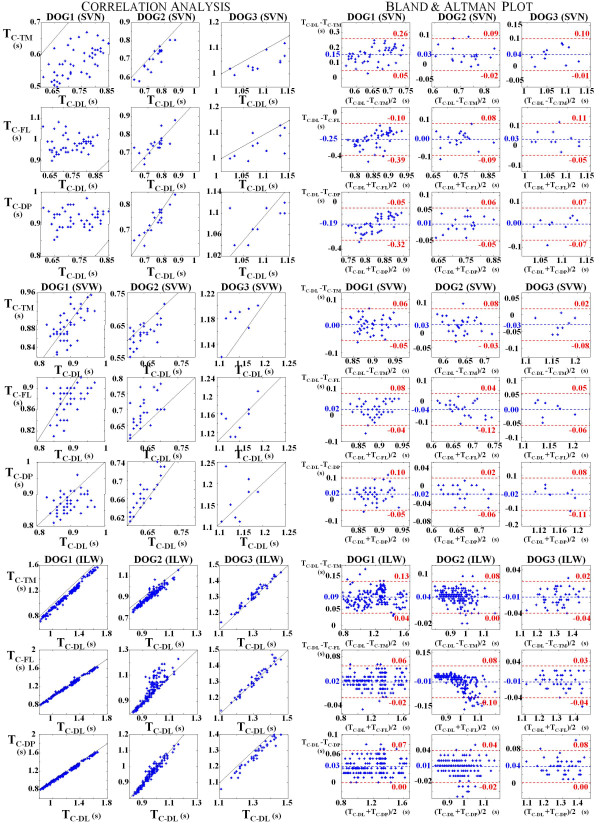
**Relationships and Bland and Altman analysis. **Relationship and Bland and Altman plot of the contraction period estimated with thoracic motion (TC-TM), respiratory airflow (TC-FL) and transdiaphragmatic pressure (TC-DP) signals versus contraction period estimated with diaphragmatic length signal (TC-DL), for the three dogs in the spontaneous ventilations without anaesthesia (SVN), spontaneous ventilations with anaesthesia (SVW) and inspiratory load with anaesthesia (ILW) respiratory tests. The solid black continuous line is the identity function (desired relationship). Each dot represents a respiratory cycle.

**Figure 4:**
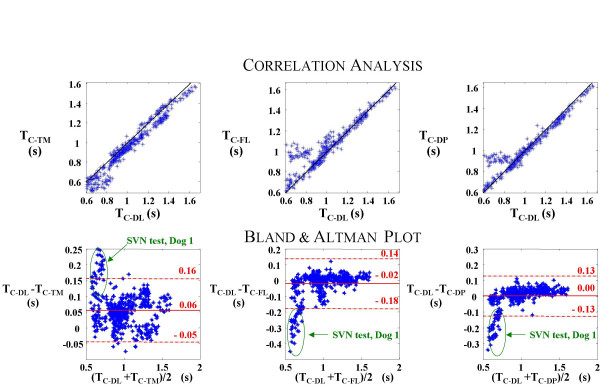
**Relationships and Bland and Altman analysis (all cycles together). **Relationship and Bland and Altman plot of the contraction period estimated with thoracic motion (TC-TM), respiratory airflow (TC-FL) and transdiaphragmatic pressure (TC-DP) signals versus contraction period estimated with diaphragmatic length signal (TC-DL), for all the respiratory cycles analyzed in the three dogs and the three respiratory tests. The solid black continuous line is the identity function (desired relationship). Each dot represents a respiratory cycle. The cycles corresponding to the spontaneous ventilation without spinal anaesthesia respiratory test of the Dog 1 are encircled in the Bland and Altman plot.

The obtained results of the comparison of contraction period estimated with the DL signal (a direct measure of diaphragm shortening) with the contraction periods estimated with the TM, FL and DP signals, are shown in Tables 2, 3 and 4, and in Figs. 3 and 4.

**Table 2:** Differences between contraction period measured with diaphragmatic length (DL) and contraction period measured with thoracic motion (TM), respiratory airflow (FL) and transdiaphragmatic pressure (DP).

	**DL vs. TM**				**DL vs. FL**				**DL vs. DP**			
	
	*MEAN (s)*	*SD(s)*	*MEAN - 2SD (s)*	*MEAN + 2SD (s)*	*MEAN (s)*	*SD(s)*	*MEAN - 2SD (s)*	*MEAN + 2SD (s)*	*MEAN (s)*	*SD(s)*	*MEAN - 2SD (s)*	*MEAN + 2SD (s)*
**Dog 1 (SVN)**	0.15	0.05	0.05	0.26	-0.25	0.08	-0.39	-0.01	-0.19	0.07	-0.32	-0.05
**Dog 2 (SVN)**	0.03	0.03	-0.02	0.09	-0.00	0.04	-0.09	0.08	0.01	0.03	-0.05	0.06
**Dog 3 (SVN)**	0.04	0.03	-0.01	0.10	0.03	0.04	-0.05	0.11	-0.00	0.04	-0.07	0.07
**Dog 1 (SVW)**	-0.00	0.03	-0.05	0.06	0.02	0.03	-0.04	0.08	0.02	0.04	-0.05	0.10
**Dog 2 (SVW)**	0.03	0.03	-0.03	0.08	-0.04	0.04	-0.12	0.04	-0.02	0.02	-0.06	0.02
**Dog 3 (SVW)**	-0.03	0.02	-0.08	0.02	-0.00	0.03	-0.06	0.05	-0.02	0.05	-0.11	0.08
**Dog 1 (ILW)**	0.09	0.02	-0.04	0.13	0.02	0.02	-0.02	0.06	0.03	0.02	-0.00	0.07
**Dog 2 (ILW)**	0.04	0.02	-0.00	0.08	-0.01	0.05	-0.10	0.08	0.01	0.02	-0.02	0.04
**Dog 3 (ILW)**	-0.01	0.02	-0.04	0.02	-0.01	0.02	-0.04	0.03	0.04	0.02	-0.00	0.08

MEAN: Mean difference; SD: standard deviation; MEAN ± 2SD: limits of agreement of the Blond and Altman analysis; SVN: spontaneous ventilation without anaesthesia respiratory test, SVW: spontaneous ventilation with anaesthesia respiratory test; ILW: inspiratory load with anaesthesia respiratory test.

**Table 3:** Correlation coefficients between contraction period measured with diaphragmatic length (DL) and contraction period measured with thoracic motion (TM), respiratory airflow (FL) and transdiaphragmatic pressure (DP).

	**DL vs. TM**				**DL vs. FL**				**DL vs. DP**			
	
	***r***	R	p	*ρ*	***r***	R	p	*ρ*	***r***	R	p	*ρ*

**Dog 1 (SVN)**	0.61	-0.44	0.43	0.58	0.12	-0.82	0.71	0.23	0.14	-0.74	0.059	0.15
**Dog 2 (SVN)**	0.91	0.77	1.00	0.84	0.72	0.73	0.65	0.81	0.90	0.89	0.76	0.87
**Dog 3 (SVN)**	0.77	0.39	0.68	0.79	0.63	0.45	0.72	0.65	058.	0.56	0.38	0.53
**Dog 1 (SVW)**	0.62	0.62	0.60	0.64	0.54	0.39	0.42	0.55	0.37	0.23	0.32	0.42
**Dog 2 (SVW)**	0.69	0.47	0.80	0.66	0.51	0.15	0.74	0.51	0.85	0.69	0.94	0.83
**Dog 3 (SVW)**	0.60	0.23	0.55	0.58	0.59	0.62	0.71	0.56	0.26	0.24	0.46	0.32
**Dog 1 (ILW)**	0.99	0.92	0.99	0.99	1.00	0.99	1.00	0.99	1.00	0.98	0.99	0.99
**Dog 2 (ILW)**	0.97	0.79	1.04	0.96	0.91	0.85	1.31	0.93	0.98	0.97	1.00	0.96
**Dog 3 (ILW)**	0.98	0.97	0.94	0.98	0.98	0.97	1.01	0.97	0.97	0.86	0.99	0.97

r: Pearson correlation coefficient; R: reliability coefficient; p: slope of the linear regression line; *ρ*: Spearman's rank-order correlation coefficient; SVN: spontaneous ventilation without anaesthesia respiratory test, SVW: spontaneous ventilation with anaesthesia respiratory test; ILW: inspiratory load with anaesthesia respiratory test.

**Table 4:** Differences and correlation coefficients between contraction period measured with diaphragmatic length (DL) and contraction period measured with thoracic motion (TM), respiratory airflow (FL) and transdiaphragmatic pressure (DP), for all the respiratory cycles analysed.

	***r***	***R***	***p***	***ρ***	*MEAN(s)*	*SD(s)*	*MEAN - 2SD (s)*	*MEAN + 2SD (s)*
**DL vs TM^1^**	0.98	0.95	1.01	0.98	0.055	0.050	-0.045	0. 156
**DL vs FL^1^**	0.94	0.93	0.86	0.91	-0.021	0.080	-0. 180	0. 138
**DL vs DP^1^**	0.96	0.96	0.86	0.94	0.001	0.064	-0. 127	0. 129
**DL vs TM^2^**	0.99	0.96	0.96	0.98	0.044	0.040	-0.032	0.125
**DL vs FL^2^**	0.99	0.99	0.97	0.98	-0.001	0.040	-0.080	0.077
**DL vs DP^2^**	0.99	0.99	0.94	0.99	0.018	0.026	-0. 034	0.070

^1^Including all the respiratory cycles analysed; ^2^Without the respiratory cycles corresponding to the spontaneous ventilations without anaesthesia test. r: Pearson correlation coefficient; R: reliability coefficient; p: slope of the linear regression line; *ρ*: Spearman's rank-order correlation coefficient; MEAN: Mean difference; SD: standard deviation; MEAN ± 2SD: limits of agreement of the Blond and Altman analysis; SVN: spontaneous ventilation without anaesthesia respiratory test, SVW: spontaneous ventilation with anaesthesia respiratory test; ILW: inspiratory load with anaesthesia respiratory test.

In the Fig. 3 is shown graphically the relationship among the periods of contraction obtained by means the different estimation methods, for each animal and each respiratory test. This relationship is showed in two formats: a plot of the data with the line of equality (all points would lie in this line), and a Bland and Altman plot [12] with the mean difference and agreement limit lines.

**Figure 5:**
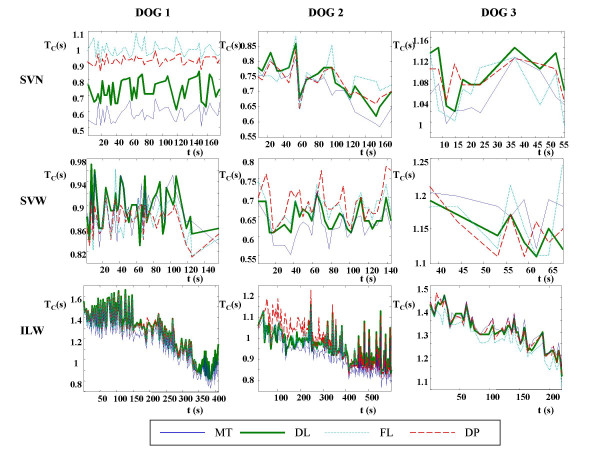
**Diaphragmatic contraction period monitoring. **Contraction period (TC) estimated with thoracic cage motion (MT: thin solid line), diaphragm length (DL: thick solid line), respiratory airflow (FL: dotted line) and transdiaphragmatic pressure (DP: dashed line) signals versus duration of the respiratory tests in seconds, for the three dogs in the spontaneous ventilations without anaesthesia (SVN), spontaneous ventilations with anaesthesia (SVW) and inspiratory load with anaesthesia (ILW) respiratory tests.

In Table 2, are shown the values of the mean difference (MEAN), the SD of the difference, and the agreement limits of the Bland and Altman plots. The mean error obtained with the three indirect measures of diaphragm shortening were lower than 0.1 seconds and the SD of the difference was lower than 0.05 seconds, except in the SVN test of Dog 1 (in this test it has been observed a great variability in the values obtained by means the four signal analyzed, as is could be seen in the first graph of Fig. 5). Furthermore the Bland and Altman agreement limits are always lower than 0.13 seconds.

Table 3 shows values of the correlation coefficient (r), the reliability coefficient (R), the slope of the linear regression line (p), and the Spearman's rank correlation coefficient between DL and TM contraction periods, between DL and FL contraction periods, and between DL and DP contraction periods for the three dogs for SVN, SVW and ILW respiratory tests. The relationships in the ILW respiratory test were nearly linear (r > 0.91), with reliability coefficients indicating a high reliability in the measurements (R > 0.79), slopes of the linear regression line very close to equality line (except in the flow of the second dog), and Spearman rank-order coefficient showing a strong link between the variables analyzed (r > 0.93).

In the SVN and SVW respiratory tests (except for the SVN test of the first dog), results showed a moderate relationship, but, in general, correlation coefficients estimated in TM signal were better than estimations in FL and DP signal. Relationship between the contraction period estimated with TM signal and contraction period estimated with DL signal showed Pearson correlation coefficients between 0.60 and 0.91, reliability coefficients between 0.23 and 0.77, slope of the linear regression lines between 0.55 and 1, and Spearman's rank-order correlation coefficient between 0.58 and 0.84.

In Fig. 4 and Table 4 are shown the results obtained analyzing all the respiratory cycles together. In both correlation analysis and Bland-Altman plots it is seen that respiratory cycles corresponding to the SVN test of Dog 1 (marked with a circle in Fig. 4) have different behaviour than the rest. For that reason in the first 3 rows of Table 4 it are shown the parameters obtained with all the cycles and in the last 3 rows are shown the parameters obtained excluding the respiratory cycles of the SVN test of the first dog. Excluding these respiratory cycles, the contraction period estimated with the TM signal tends to give a lower reading than the measure made with the DL signal, with a mean of 0.04 seconds (0.06 without the exclusion), a standard deviation of 0.04 seconds (0.05 without the exclusion), and limits of agreement between -0.03 and 0.12 seconds (between -0.05 and 0.16 without the exclusion). These results are slightly worse than the results obtained from the comparison with the FL signal or the DP signal. The correlation coefficients are very similar in the three signals.

Finally, Figure 5 shows the evolution of the diaphragm contraction periods estimated with the four signals throughout the SVN, SVW and ILW respiratory tests for the three dogs studied. It is seen that, in all cases, the behaviour of contraction time estimated with the four signals is very similar (although the MEAN difference of the SVN test of the first dog is unsatisfactorily great, as seen in Table 2).

## Discussion

In the present work we have compared the rib cage motion recorded by surface sensors with the changes in diaphragm activity registered by sonomicrometry, transdiaphragmatic pressure and airflow recorded in dogs during spontaneous and inspiratory load breathing. Diaphragmatic time contraction measured with a surface sensor has a good correlation with the rest of signals, especially during the inspiratory load test.

In a recent study we observed that the beginning and the end of diaphragmatic contraction were indicated with inflexion points in the thoracic cage motion signal, acquired with a piezoelectric contact sensor placed on the costal wall of the thorax [6,7]. An algorithm was implemented and validated to detect the initial and final instants of diaphragm contraction, and the results were compared with the direct measurement of the diaphragmatic muscle length changes made by sonomicrometry [8]. In the present work we have compared the contact sensor signal with other transducer signals to test the monitoring capacity of contact sensors in different respiratory patterns. Three different tests have been studied: spontaneous ventilation before the use of spinal anaesthesia, spontaneous ventilation (SVW) after spinal anaesthesia, and breathing through a resistive inspiratory load (ILW).

Spinal anaesthesia was used in the present study as a means to isolate diaphragmatic activity by eliminating the activity of the intercostals muscles. In this way, TM, FL and DP signals are directly related with the contraction of diaphragm muscle in the SVW and ILW respiratory tests (as well as DP signal). In the SVN test the morphologies of TM, FL and DP signals are influenced by the activity of intercostal muscles. However, time differences between contraction periods measured in SVN and SVW respiratory tests were very similar (except for the SVN test of the first dog). This could be explained taking into account that during spontaneous breathing the activity of intercostals and respiratory accessory muscles is minimal [6,7].

A close relationship between the different methods of diaphragm contraction time estimation has been found during the inspiratory load test. However, during spontaneous ventilation, the correlation was lower than that obtained in the resistive load test. This difference could be explained by the fact that during spontaneous ventilation, the motion of the respiratory system is very low and in consequence, the signal recorded by the contact sensor (and in general, all the respiratory signals) has not sufficient intensity to detect with the same precision the beginning and ending of diaphragmatic activity. Besides, during inspiratory resistive loading, there is a marked distortion of the rib cage (in particular when the intercostal muscles are paralyzed) and this produces a more marked thoracic-cage motion signal.

Another important finding of this work was that time contraction differences between signals were less than 0.1 s for spontaneous ventilation and inspiratory load (except in the irregular case of the SVN respiratory test of the first dog), which indicates that the application of contact sensors constitutes an indirect way to detect the diaphragmatic activity. This could be seen in the graphs of Fig. 5, in which we observed that practically in all cases the behavior of the diaphragmatic contraction time estimated with the four signals is very similar, being appropriate for diaphragmatic contraction period monitoring. The TM signal has the advantage with respect to other signals in that it is non-invasive and does not affect the breathing pattern [2-4]. Therefore, it is suitable for continuous monitoring of breathing pattern parameters such as respiratory frequency and diaphragmatic contraction time (which is very close to inspiratory time). Thus, the usefulness of TM signal for non-invasive diaphragmatic contraction period monitoring is demonstrated. A particular case is the SVN test of the first dog. In this case the breathing was very weak, causing that beginning of the contraction was very slow and irregular. This generated difficulties to determine the beginning of the contraction, provoking great differences in the contraction time estimated with the four signals. Nevertheless, the evolution of contraction time throughout the respiratory tests is very similar.

The contraction of the costal diaphragm acts to displace the rib cage through its insertions at the costal margins and by changing the pressure on the inner surface of the rib cage in the area of apposition. The crural diaphragm is not inserted on the rib cage, but it is considered to have an action through the central tendon. Therefore, in spontaneous respiration the crural part has an important inflationary action on the lower rib cage [13], and although we only registered costal muscle length changes, we believe that the contact sensor shows the activity of both diaphragm components.

Finally, to acquire the diaphragm shortening signal, it has been necessary to surgically isolate their diaphragms. The effect of diaphragm isolation could be to favour the operation of the piezoelectric contact sensor to measure diaphragmatic contraction timing. This should be kept in mind when extrapolating these results to human.

## Conclusions

The technique presented in this work represents a non-invasive method to assess the timing of diaphragm contraction in dogs. We believe that in the future this technique could provide a new potentially useful method for non-invasive respiratory timing monitoring in humans.

## Competing interests

None declared

## Authors' contributions

JAF, JM, RJ and AG conceived the study, and participated in its design and coordination. JAF and AG designed and conducted the experiments. AT participated in the design of the study and performed the signal processing and statistical analysis. BG and JG provided advice on analysis of the data and manuscript writing. JAF, AT and RJ wrote the first draft of this manuscript. All authors read and approved the final manuscript.

## Pre-publication history

The pre-publication history for this paper can be accessed here:


